# Anaesthetic emergence agitation in adults following general surgery: A scoping review

**DOI:** 10.1016/j.ijnsa.2025.100320

**Published:** 2025-03-18

**Authors:** Meredith Heily, Marie Gerdtz, Rebecca Jarden, Yen Yap, Rinaldo Bellomo

**Affiliations:** a Faculty of Medicine, Dentistry & Health Sciences, University of Melbourne, Melbourne, Australia; b The Royal Melbourne Hospital, Melbourne, Australia; c Austin Health, Melbourne, Australia

**Keywords:** Agitation, Anaesthesia, Critical care, Emergence, Intensive care, Postoperative, Surgery

## Abstract

**Background:**

Anaesthetic emergence is the patient's transition from general anaesthetic until they are alert and in full control of vital reflexes. It is during this transition that significant complications, including anaesthetic emergence agitation, may occur. A preliminary search did not identify any research investigating adults who undergo anaesthetic emergence in critical care settings.

**Objectives:**

To map the post-anaesthetic literature reporting outcomes, risk factors, and management of adult patients admitted directly to a critical care unit, who develop emergence agitation, and to describe the implications for clinical practice.

**Methods:**

The scoping review was registered at https://osf.io/spwx5/ and conducted using the Joanna Briggs Institute methodology, with the framework of Population, Concept and Context. Search terms including agitation, anaesthetic, emergence, postoperative and surgery.

**Results:**

Twenty-five articles were identified and were eligible for data extraction. Risk factors included co-morbid conditions, anaesthetic agents and the presence of in-situ invasive devices. Studies varied regarding design and patient assessment tools. Data were reported from one or more of eight observation timepoints along the emergence continuum, from end-anaesthetic until post anaesthetic care unit discharge. No studies investigated patients with direct postoperative admission to critical care settings.

**Conclusions:**

This review has characterised the emergence continuum. The variations between studies has highlighted the necessity to reach future consensus regarding emergence definition and measurement. A critical gap was identified regarding recommendations for prevention and management of emergence agitation for patients admitted directly to a critical care unit.

## What is already known

• Anaesthetic emergence agitation following surgery presents several risks, such as a higher chance of injury, self-extubation, bleeding, and extended hospital stays.
• Risk factors include emergency surgery, cognitive disorders, severe co-morbid conditions, duration of surgery, and presence of in-situ invasive devices at emergence.
• Pharmacological and non-pharmacological measures such as selection of chemical anaesthetic agents and preoperative patient risk assessment decrease the risk of emergence agitation.

## What this paper adds

• The anaesthetic emergence continuum has been characterised from the end of anaesthesia to post anaesthetic care unit discharge.
• There are variations in study designs, characterisation of emergence agitation and data collection methods, indicating that published evidence needs to be interpreted in light of these variations.
• Little is known about the outcomes of emergence agitation and the best way to manage the problem in patients directly admitted to a critical care unit following surgery.

## 1 Background

Anaesthetic emergence is defined as the time between cessation of the anaesthetic (end of anaesthesia) and the time when the patient is alert and in full control of their airway and vital reflexes (Foulds and Dalton, 2018). It is during this timeframe that the patient is highly vulnerable to complications such as clinical instability, airway compromise (Klein et al., 2021), and anaesthetic emergence agitation (Choi et al., 2015).

Anaesthetic emergence agitation, sometimes also referred to as emergence delirium (Lepouse et al., 2006), has been characterised as an acute and fluctuating alteration of mental state that manifests as agitation, confusion, disorientation, and possible combative behaviour (Yu et al., 2010). Severe emergence agitation has been associated with adverse events, including bleeding, self-extubation, and removal of catheters and drain tubes (Wilson and Pokorny, 2012). In such situations, management to maintain patient safety may require involvement of up to six times more nursing staff than for non-agitated patients (Yu et al., 2010). Emergence agitation has been reported more often in children than adults (Azemati et al., 2013). This observation may be due to a range of factors including age, type of surgery performed, airway obstruction, unfamiliar environment at the time of emergence, post-operative pain, and individual psychological factors (Aouad and Nasr, 2005).

In most surgical procedures, patients emerge from anaesthesia and have their invasive airway device (such as the laryngeal mask or endotracheal tube) removed in the operating theatre (Australian and New Zealand College of Anaesthetists, 2020). After their surgery, patients are routinely recovered in the postanaesthetic care unit (Card et al., 2015).

In contrast, patients who are critically unwell, require complex care, or are potentially unstable due to major surgery or co-morbidities, may be transferred to other post-operative settings to be recovered (Austin Health Postoperative Surveillance Team (Post), 2010). Many of these patients arrive in settings such as high dependency or critical care environments still under general anaesthesia, with an endotracheal tube in situ and connected to mechanical ventilation (Stephens and Whitman, 2015). Anaesthetic emergence will be initiated and completed by critical care staff (doctors and nurses) when the individual is clinically ready, which is often shortly after admission (Gutsche et al., 2014).

A preliminary search of the published literature did not identify any publications reporting on anaesthetic emergence outcomes, risk factors and management for adults who were admitted directly from the operating theatre to critical care units. Hence, the preliminary search was widened to include all locations such as the operating theatre and post anaesthetic care unit. Using this process, three systematic reviews (Wei et al., 2021, Yang et al., 2023, Zhang et al., 2019) and two narrative reviews (Lee and Sung, 2020, Tolly et al., 2021) were identified. These reviews did not report findings for patients who were admitted directly to a critical care unit where they underwent anaesthetic emergence prior to removal of mechanical ventilation and their tracheal tube. All five reviews reported that patients with significant co-morbidities and risk factors were at a higher risk of emergence agitation. The risk factors included age, male gender, smoking/substance misuse (Wei et al., 2021), inhalational anaesthetic rather than propofol (Yang et al., 2023) and the omission of dexmedetomidine during anaesthetic (Zhang et al., 2019). These risk factors can be found in many patients admitted to critical care units after surgery (Onwochei et al., 2020). The narrative reviews discussed the varied use of terminology, definition, management, and the post-anaesthetic timeline for the diagnosis of emergence agitation (Lee and Sung, 2020, Tolly et al., 2021). However, the implications of these risk factors for patients who undergo anaesthetic emergence in critical care units were not identified by the previous reviews.

A scoping review methodology was selected to explore the published literature. This approach was selected in response to the lack of empirical evidence identified from the preliminary review. The objectives of this review were to map the literature on outcomes, risk factors and management of adult patients who develop emergence agitation after general surgery, particularly patients with significant co-morbid conditions and/or who are admitted to a critical care unit prior to initiation of anaesthetic emergence, and to describe the implications for clinical practice.

Key review questions included:

Review Question 1. How is anaesthetic emergence agitation defined and characterised in adult patients?
Review Question 2. What are the anaesthetic emergence agitation outcomes, management, and risk factors for patients who have emergency or major surgery, significant pre-operative co-morbidities, or admission to a critical care unit?
Review Question 3. What are the gaps in the evidence regarding patients with multiple co-morbid conditions and/or admitted to a critical care unit prior to anaesthetic emergence?

## 2 Methods

### 2.1 Design

The protocol was registered with Open Science Framework (https://osf.io/spwx5/). Human Research Ethics committee approval was not required as data were sourced only from published literature. The review was conducted according to the Joanna Briggs Institute methodology for scoping reviews, (Peters et al., 2020) and the Preferred Reporting Items for Systematic Reviews and Meta-Analyses extension for Scoping Reviews (PRISMA-ScR). This methodology requires a multi-step process including developing the search concepts, search terms, and search strategy (Tricco et al., 2018).

### 2.2 Developing concepts and definitions – Population, concept and context

The multi-step strategy used a team-based approach to develop the understanding of the population of interest, develop the concept being examined and to place the findings within the context (Peters et al., 2020). The steps are needed to extract, clarify, analyse and present the diverse range of evidence that may result from a scoping review (Pollock et al., 2023). Our team consisted of the authors (MH, MG, RJ, YY, RB), and a team-based approach was used to develop, review and implement each step of the scoping review, within the framework of Population, Concept and Context (Peters et al., 2020).

*Population:* Adult patients 18 years or older, who underwent anaesthetic emergence after surgery undertaken with a general anaesthetic.
*Concept:* Studies that had a primary outcome which identified and evaluated adult patients undergoing anaesthetic emergence after invasive general surgery were considered for inclusion if they reported on one or more of the following emergence agitation topics: predictors; risk factors; complications; prevalence; management and clinical locations of studies.
*Context:* This scoping review considered studies published from 1/1/2017 – 31/12/2022 of all research designs and from all countries.

### 2.3 Data sources and search strategy

The search strategy was developed with the advice and assistance of senior librarians. Search terms were agreed upon, and a data extraction form developed. Following this, the process of screening by title and abstract, full text and data extraction occurred. The search strategy used the Population, Concept and Context format described above (Pollock et al., 2023).

### 2.4 Eligibility criteria

Studies were included if they reported on one or more outcomes directly related to anaesthetic emergence agitation which occurred after general surgery undertaken with a general anaesthetic; surgery included an open approach with an incision through skin and muscle; were published in the English language; and included adults 18 years or older.

Following testing with MEDLINE (Ovid) and SCOPUS, the search terms and synonyms were adapted for each database. The search focussed on title, abstract and key words, using concepts such as anaesthetic, surgery, postoperative, emergence, agitation and delirium (Supplementary Materials M1).

### 2.5 Exclusion criteria

Studies were excluded if they: did not involve adults undergoing a general anaesthetic and invasive general surgery; were systematic or other reviews; were systematic or other reviews that did not directly report on patients within the timeframe; only included endovascular, endoscopy, imaging, or laboratory procedures; only investigated post-operative delirium; were not published in the English language.

### 2.6 Databases and data management

Six databases, one grey literature repository, and Google Scholar were searched for studies, including ‘in press’ publications. The databases comprised MEDLINE (Ovid), SCOPUS, CINAHL (EBSCOhost), EMBASE (Ovid), Web of Science (Clarivate Analytics), and PUBMED (PubMed NCBI). Grey literature was searched through ProQuest Dissertations & Theses and Google Scholar. The search was completed on 29^th^ April 2023 (Supplementary Materials M1).

All citation information was exported from the databases to Endnote^TM^ X20 (Clarivate Analytics, PA, USA) (Endnote, 2023) then to Covidence^TM^ (Veritas Health Innovation, Melbourne, Australia) (Covidence, 2023) for study de-duplication, screening and data extraction.

### 2.7 Screening, data extraction, analysis

The authors progressively screened articles by titles and abstracts, followed by full text. Included articles then underwent data extraction using a customised form in Covidence. Primary screening was conducted by MH, with second reviews by RJ or CY. As needed, third reviewer agreement was provided by MG. In line with the aims of the scoping review to map the existing literature, a quality appraisal was not conducted on the included articles (Cacchione, 2016).

Extracted data were classified according to the customised form, with headings such as patient characteristics, type of surgery, type of anaesthetic, location where emergence was implemented, study methods and outcomes (Supplementary Materials M2).

### 2.8 Synthesis

Tables were developed to synthesise the data, analyse the results from the review, and assess the results against the scoping review questions. Assessment of preliminary outcomes was followed by secondary analysis and development of tables regarding measurement of anaesthetic emergence agitation terminology, definitions and observational timepoints along the anaesthetic emergence continuum (from cessation of general anaesthetic in the operating room until discharge from the post anaesthetic care unit) (Greiner and Kremer, 2019). Outcomes were then synthesised to establish definitional clarity or disparities, differences between studies and gaps in the published literature.

## 3 Results

**Fig. 1:**
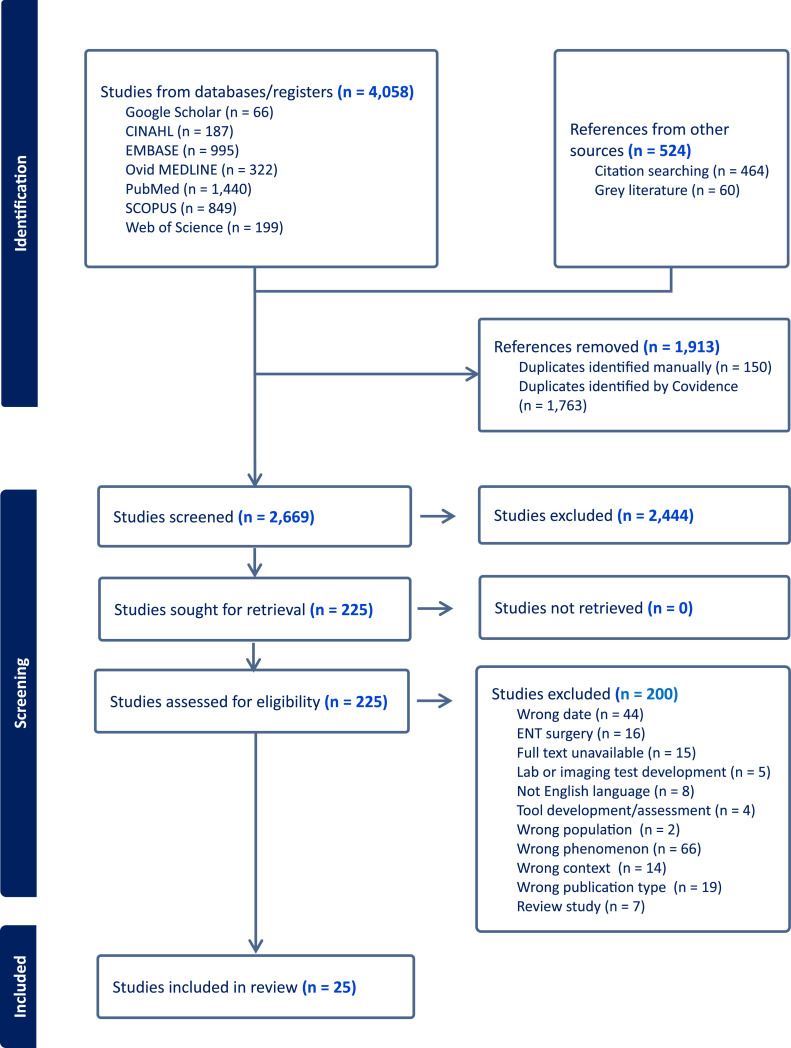
PRISMA* Flow Chart * PRISMA – Preferred Reporting Items for Systematic Reviews and Meta-Analyses *Legend: ENT – ear, nose, throat*.

The initial search identified 4,058 publications. After de-duplication, title, abstract, and full-text screening, twenty-five articles were selected and underwent data extraction. The search and screening procedure is illustrated in Fig. 1.

### 3.1 Characteristics of studies

All selected studies included adult patients in hospital settings, undergoing general surgery under a general anaesthetic. Most studies in the review recruited patients who underwent elective surgery or did not report results differentiating between which patients may have undergone emergency surgery. Enrolment of patients in the studies had specific inclusion and exclusion criteria. The criteria are detailed in sub-section 3.1, Exclusions, Table 1 and Table 3 (Table 1, Table 3).

**Table 1:** Characteristics of studies (n = 25).

**Author, Year,** **Country**	**Outcome measures, study settings**	**Design**	**EA Definition**	**Measurement**	**Population**		
					**Demographics**	**Anaesthetic**	**Study Exclusions**
**OBSERVATIONAL STUDIES**							
**Assefa & Sahile** **(2019)** **Ethiopia**	Incidence of ED in: - PACU	Prospective cross-sectional cohort	Anxious or apprehensive, movements not aggressive or vigorous	RASS ≥ +1	306 patients Age ≥ 18 years Surgery: General & includes emergency	TIVA (propofol & thiopentone)	Intubated preop Neurological/psychiatric disorders Transfer to ICU
Bharadwaj et al (2022) **India**	Incidence of EA & ED in: 1. OT 2. PACU	Prospective cohort	Anxious or mildly agitated, attempting to sit up, calms down on verbal instruction	RSAS ≥ 5	320 patients Age ≥ 18 years Surgery: Intracranial	Combination	Emergency Intubated preop Neurological/psychiatric disorders Remains intubated postop Transfer to ICU
**Braga & Abelha** **(2022)** **Portugal**	Incidence of IE & POD in: 1. PACU 2. Ward	Prospective cohort	Anxious or apprehensive, movements not aggressive or vigorous	RASS ≥ +1	148 patients Age ≥ 18 years Surgery: Cancer	Not reported	Emergency Neurological/psychiatric disorders Remains intubated postop Transfer to ICU
**Fei & Yu** **(2019)** **China**	Incidence of EA, ED & POD in: 1. OT 2. PACU 3. Ward	Prospective cross-sectional cohort * OT = End-anaesthesia to extubation	Anxious or apprehensive, movements not aggressive or vigorous	RASS ≥ +1	380 patients Mean age 56 years Surgery: Cancer	Inhalational (sevoflurane, desflurane, isoflurane)	Emergency Neurological/psychiatric disorders Remains intubated postop Transfer to ICU
**Fields et al** **(2018)** **USA**	Incidence of EA in: - PACU	Retrospective case-matched	Very agitated. Pulls on or removes tube(s) or catheter(s) or has aggressive behaviour towards staff	RASS > +3 Or Haloperidol administration	207,569 records: 510 records case-matched Surgery: General & includes emergency	All types – case matched to individual anaesthetic and patient details	Remains intubated postop Transfer to ICU
**Gu et al** **(2022)** **China**	Incidence of ED & POD in: 1. ICU 2. Ward	Prospective cohort	Anxious or apprehensive, movements not aggressive or vigorous.	RASS ≥ +1	618 patients Age ≥ 18 years Surgery: Intracranial	Chemical & inhalational	Emergency Lactating Neurological/psychiatric disorders Pregnancy Survival estimated < 24 hours
**Huang et al** **(2020)** **China**	Incidence of ED & POD in: - PACU	Prospective cohort	Inappropriate behaviour to place and/or for the person	Nu-DESC ≥ 2	168 patients Age ≥ 65 years Surgery: General	Intravenous & inhalational	Age < 65 years Cardiac sx Emergency Neurological/psychiatric disorders Organic encephalopathy Postop hypoxaemia Transfer to ICU
**Makarem et al** **(2020)** **Iran**	Incidence of IE in: 1. OT (end-anaesthetic -extbn 2. PACU	Prospective cohort	Anxious or apprehensive, movements not aggressive or vigorous	RASS ≥ +1	1,136 patients, 95 with substance abuse history Age ≥ 18 years Surgery: General	Chemical & inhalational	ASA ≥ IV Cardiac sx Emergency Intracranial sx Neurological/psychiatric disorders Transfer to ICU
**Mekonin et al** **(2022)** **Ethiopia**	Incidence of ED in: - PACU	Prospective cohort	1. Anxious or apprehensive, movements not aggressive or vigorous 2. Positive for delirium	1. RASS ≥ +1 2. CAM-ICU	384 patients Age ≥ 60 years Surgery: General	Variable between patients	Age < 60 years Complicated co-morbid diseases Emergency Intracranial sx Neurological/psychiatric disorders Transfer to ICU Unconscious in PACU
**Pipanmekaporn et al** **(2018)** **Thailand**	Incidence of ED in: - PACU	Prospective cohort	Anxious or apprehensive, movements not aggressive or vigorous	RASS ≥ +1	2,206 incident forms All age groups. Surgery: General & includes emergency	Variable between patients	Transfer to ICU
Ramroop et al (2019) **Trinidad & Tobago**	Incidence of ED in: - PACU	Prospective cohort	Inappropriate behaviour to place and/or for the person	Nu-DESC > 2	417 patients Age ≥ 18 years Surgery: General Elective & Emergency	Inhalational (sevoflurane)	Emergency Neurological/psychiatric disorders Remains intubated Transfer to ICU
Wiinholdt et al (2019) **Denmark**	Incidence of IE in: - PACU	Prospective cohort	Inappropriate communication to place and/or for the person	Nu-DESC > 2	1,000 patients Age ≥ 12 years Surgery: abdominal or orthopaedic	Chemical, inhalational, combined	Neurological/psychiatric disorders Non-business hours Remains intubated Transfer to ICU
Zhang et al (2020) **China**	Incidence of EA, ED & POD in: 1. OT 2. PACU 3. Ward	Prospective cohort	1. Anxious or apprehensive, movements not aggressive or vigorous 2. Positive for delirium	1. RASS ≥ +1 2. CAM-ICU	915 patients Age 65 – 90 years Surgery: Major non-cardiac	Chemical and inhalational	Age < 65 years ASA ≥ IV Emergency Neurological/psychiatric disorders Remains intubated Transfer to ICU
**RANDOMISED CONTROLLED TRIALS**							
**Awada et al** **(2022)** **Denmark**	Incidence of ED & POD in: - PACU	Prospective RCT – High dose vs Low dose steroids	Positive for delirium	3D-CAM positive	53 patients Age > 18 years Surgery: liver	Combined	Allergy to study drug Combined surgery Emergency Epidural not feasible Insulin dependent diabetes Pregnancy or lactation Recent steroids Transfer to ICU
**Cho et al** **(2021)** **Korea**	Incidence of EA in: 1. OT (End-anaesthesia to extubation) 2. PACU	Prospective RCT – Placebo VS Steroids	Anxious or mildly agitated, attempting to sit up, calms down on verbal instructions.	RSAS ≥ 5	90 patients Age 19 – 80 Surgery: urological with postoperative IDC	Chemical & inhalational	Active infection Age > 80 years old Anaesthesia > 6 hours ASA ≥ IV Bladder/prostate abnormalities End-stage renal disease Immunosuppressed Neurological/psychiatric disorders Uncontrolled diabetes
**Choi et al** **(2021)** **Korea**	Incidence of EA in: 1. OT (during extubation) 2. PACU	Prospective RCT – Placebo vs Dexmed	Anxious or mildly agitated, attempting to sit up, calms down on verbal instructions	RSAS ≥ 5	88 patients Age 18 – 75 years Surgery: Laparoscopic cholecystectomy	Chemical & inhalational + Adjunct (study drug)	Age < 20 years old Age > 75 years old Allergy to study drug ASA ≥ III Emergency Neurological/psychiatric disorders Pregnancy Transfer to ICU
**Kang et al** **(2019)** **China**	Incidence of EA in: - PACU	Retrospective Cohort Comparison – Control vs Dexmed	Anxious or mildly agitated, attempting to sit up, calms down on verbal instructions	RSAS ≥ 5	2,468 patient records Age ≥ 18 years Surgery: lung cancer	Combined + Adjunct (study drug)	Emergency Neurological/psychiatric disorders Reoperation Transfer to ICU
Kawagoe et al (2022) **Japan**	Incidence of EA in: 1. OT (end-anaesthesia to extubation) 2.PACU	Prospective RCT – Desflurane vs propofol	Anxious or apprehensive, movements not aggressive or vigorous	RASS ≥ +1	80 patients Age 20 – 75 years Surgery: lung cancer	Epidural + Desflurane (inhalational) OR Epidural + propofol (chemical)	Active inflammation Age < 20 years Age > 75 years ASA ≥ III Cardiac disease NYHA > II Emergency Epidural not feasible Neurological/psychiatric disorders Pneumonectomy Pulmonary hypertension Respiratory disorders Severe cardiac disease Steroids/similar < 3 months Transfer to ICU
**Kim et al** **(2019)** **Korea**	Incidence of EA & POD in: 1. OT (post-extubation) 2. PACU 3. ICU or ward	Prospective RCT – Dexmed vs placebo	Anxious or mildly agitated, attempting to sit up, calms down on verbal instructions	RSAS ≥ 5	120 patients Age 18 – 75 years Surgery: VATS	Chemical & inhalational + Adjunct (study drug)	Age > 75 years old Allergy to study drug Anti-arrythmia medications ASA > III BMI > 30 kg/m^2^ Bradycardia < 50/min Cardiac arrythmia Cardiac disease NYHA > II Cardiac ejection fraction < 40% Chronic pain Drug addiction Emergency Neurological/psychiatric disorders Raised liver/kidney serum enzymes
Kong et al (2021) **China**	Incidence of EA in: - post extubation (location not reported)	Prospective RCT – Dexmed: high, medium or low dose groups	Behavioural responses such as moving hands and feet	3PS > 2	180 patients Age – Not reported. Surgery: radical colorectal cancer	Chemical + Adjunct (study drug):	Abdominal skin infection Anticoagulation < 1 month ASA ≥ III Allergy to study drug Emergency Neurological/psychiatric disorders Organic diseases Surgical contraindications
Liu et al (2022) **China**	Incidence of EA in: - OT	Prospective RCT – intercostal nerve blocks vs paravertebral nerve blocks	Moderately agitated or restless	4PS ≥ 3	97 patients Age 55-75 years Surgery: VATS lobectomy	Combined + Regional block	Acute/chronic pain Age < 55 years old Age > 75 years old Alcohol/substance abuse ASA ≥ III BMI ≥ 40 kg/m^2^ Cardiopulmonary disease Coagulopathies Emergency Hepatorenal disease Narcotic use Neurological/psychiatric disorders Transfer to ICU
**Meng et al** **(2022)** **China**	Incidence of EA in: 1. OT (post extubation) 2. PACU	Prospective RCT – Butorphanol vs placebo	Anxious or mildly agitated, attempting to sit up, calms down on verbal instructions	RSAS ≥ 5	602 patients Age 18 – 70 years Surgery: VATS lobectomy	Combined + Adjunct (study drug)	Age > 70 years old ASA > III BMI > 30 kg/m^2^ Cardiac ejection fraction < 40% Emergency Heart failure Neurological/psychiatric disorders Severe hepatic dysfunction Renal dialysis Transfer to ICU
Sirivanasandha et al (2018) **Thailand**	Incidence of EA in: 1. OT (during and post extubation) 2. PACU	Prospective RCT – Dexmed vs placebo	Anxious or mildly agitated, attempting to sit up, calms down on verbal instructions	RSAS ≥ 5	98 patients Age 18 – 70 years Surgery: Anterior Cervical Discectomy and Fusion	Chemical & inhalational + Adjunct (study drug)	Abnormal liver/renal function Age> 70 years old Allergy to Dexmed/opioids ASA > III Beta blockers BMI > 30 kg/m^2^ Bradycardia Cardiac diseases Emergency Preop and postop unstable haemodynamics Remains intubated Transfer to ICU
Sun et al (2022) **China**	Incidence of EA in: - PACU	Prospective RCT – Dexmed vs placebo	Moderately agitated or restless.	4PS ≥ 3	80 patients Age > 65 years Surgery: Radical cancer Surgery duration > 2 hours	Chemical & inhalational + Adjunct (study drug)	Age < 65 years old Alcohol abuse Allergy to study drug ASA > III BMI < 28 kg/m^2^ Bradycardia Chronic pain 60 mmHg < DPB > 180 mmHg Difficult airway anatomy Emergency Malignant hyperthermia Neurological/psychiatric disorders SBP > 180 mmHg Severe organ disease Severe infection Surgery < 2 hours Transfer to ICU
**Zhang** **(2021)** **China**	Incidence of ED in: - PACU	Prospective comparison study – Nourished vs Malnourished patients	Anxious or apprehensive, movements not aggressive or vigorous	RASS ≥ +1	915 patients Age 65 – 90 years Surgery: non-cardiac	Chemical & inhalational	Age < 65 years old Age > 90 years old Anaesthesia < 2 hours ASA ≥ IV Emergency surgery Neurological/psychiatric disorders Transfer to ICU

Note: < is less than; > is greater than; ≥ is greater than or equal to

Legend: 3-D-CAM – Three Minute Confusion Assessment Method; 3PS – Three Point Scale; 4PS – Four Point Score (Aono's); ASA – American Society of Anesthesiologists Physical Status; BMI – Body Mass Index; BZD – Benzodiazepines; CAM – Confusion Assessment Method; CI – Confidence Interval; Dexmed – Dexmedetomidine; DPB – Diastolic Blood Pressure; EA – Emergence Agitation; ED – Emergence Delirium; GA – General Anaesthetic; GCS – Glasgow Coma Scale; ICU – Intensive Care Unit; IE – Inadequate Emergence; Intvn – Intervention; Intubated – tracheal intubation; LOS – Length of Stay; Nu – DESC – Nurses Delirium Screening Scale; OR – Odds Ratio; OT – Operating Theatre; PACU – Post Anaesthetic Care Unit; POD – Postoperative Delirium; RASS – Richmond Agitation Sedation Scale; RCT – Randomised Controlled Trial; RSAS – Riker Sedation Agitation Scale; SBP – Systolic Blood Pressure; sx – surgery; TIVA – Total Intravenous Anaesthetic; VATS – Video Assisted Thorascopic Surgery; vs – versus

Ten studies were from China (Fei and Yu, 2019, Gu et al., 2022, Huang et al., 2020, Kang et al., 2019, Kong et al., 2021, Liu et al., 2022, Meng et al., 2022, Sun et al., 2022, Zhang et al., 2021, Zhang et al., 2020); three from The Republic of Korea (Cho et al., 2022, Choi et al., 2021, Kim et al., 2019); two from Ethiopia (Assefa and Sahile, 2019, Mekonin et al., 2022); two from Denmark (Awada et al., 2022, Wiinholdt et al., 2019); two from Thailand (Pipanmekaporn et al., 2018, Sirivanasandha et al., 2018); and one each from India, (Bharadwaj et al., 2022) Trinidad & Tobago (Ramroop et al., 2019), Japan (Kawagoe et al., 2022), Portugal (Braga and Abelha, 2022), the United States of America (Fields et al., 2018), and Iran (Makarem et al., 2020).

Study designs were either observational (Assefa and Sahile, 2019, Bharadwaj et al., 2022, Braga and Abelha, 2022, Fei and Yu, 2019, Fields et al., 2018, Gu et al., 2022, Huang et al., 2020, Makarem et al., 2020, Mekonin et al., 2022, Pipanmekaporn et al., 2018, Ramroop et al., 2019, Wiinholdt et al., 2019, Zhang et al., 2020) or randomised controlled trials (Awada et al., 2022, Cho et al., 2022, Choi et al., 2021, Kang et al., 2019, Kawagoe et al., 2022, Kim et al., 2019, Kong et al., 2021, Liu et al., 2022, Meng et al., 2022, Sirivanasandha et al., 2018, Sun et al., 2022, Zhang et al., 2021). Prospective studies ranged in sample size from 53 patients (Awada et al., 2022) to 2,206 (Pipanmekaporn et al., 2018). Retrospective studies, where patient records were reviewed, ranged from 2,468 (Kang et al., 2019) to 207,569 (Fields et al., 2018) (Table 1).

*Review Question 1. How is anaesthetic emergence agitation described and characterised in adult patients?*

### 3.2 Anaesthetic emergence continuum

This review ascertained eight discrete timepoints in which data were reported by the studies within this scoping review. We expanded the concept of the emergence continuum to include the commencement of the emergence continuum in the operating theatre, progressing to admission to the post anaesthetic care unit as described by Greiner and Kremer (2019).

The anaesthetic emergence continuum commences at the time of the end of anaesthesia and ceases at the point of discharge from the post anaesthetic care unit. The transition timepoints observed are: 1 = end anaesthesia, 2 = pre-extubation, 3 = tracheal extubation, 4 = within five minutes of extubation, 5 = on admission to the post anaesthetic care unit, 6 = 30 minutes after admission to the post anaesthetic care unit, 7 = 60 minutes after post anaesthetic care unit admission, 8 = pre-discharge to the ward. Timepoint 9 = postoperative delirium ward assessment after postanaesthetic care unit discharge (Awada et al., 2022, Bharadwaj et al., 2022, Gu et al., 2022, Kim et al., 2019, Zhang et al., 2020) (Table 2).

**Table 2:** Anaesthetic emergence continuum: observation timepoints and terminology (n = 25).

**Term used**	**Operating Theatre**				**Post Anaesthetic Care Unit**													*Ward*
	End anaes	Pre- extubn	Extubn	5 mins extubn	O/A			≤ 30 mins			60 mins				Discharge			
	***EA***	***EA***	***EA***	***EA***	***EA***	***ED***	***POD***	***EA***	***ED***	***POD***		***EA***	***ED***	***POD***	***EA***	***ED***	***POD***	***POD***
Assefa (2019)						√			√									
Awada (2022)									√							√		√
Bharadwaj (2022)				√					√				√					√
#Braga (2022)						√										√		
Cho (2022)	√	√	√	√	√			√				√			√			
Choi (2021)			√			√												
Fei (2019)			√			√				√			√			√		
Fields (2018)					√			√				√			√		√	
Gu et (2022)						√												√
Huang (2020)									√				√					
Kang (2019)				√														
Kawagoe (2022)			√	√	√			√				√						
Kim (2019)			√	√	√			√				√			√			√
+Kong (2021)					√													
Liu et (2022)	√	√	√															
Makarem (2020)								√										
Mekonin (2022)						√			√									
Meng (2022)				√														
Pipanmekaporn (2018)						√			√				√			√		
Ramroop (2019)									√									
Sirivanasandha (2018)		√	√	√				√										
Sun (2022)					√			√										
#Wiinholdt (2019)						√			√							√		
Zhang (2021)						√			√									
Zhang (2020)			√			√			√				√			√		√

Legend:+ Location not specified; #Used the phrase ‘Inadequate emergence’, of which the agitated component was called emergence delirium

EA – emergence agitation; ED – emergence delirium; End anaes – end anaesthetic; Extubn – tracheal extubation; Mins- minutes O/A – on admission; OT – operating theatre; PACU – post anaesthetic care unit; POD – postoperative delirium; pre-extubn – immediately prior to tracheal extubation; 5 min extubn – five minutes after tracheal extubation; ≤ 30 min – within 30 minutes

### 3.3 Variable terminology and timepoints

Variation was observed at data collection regarding terminology used to name emergence agitation, clinical location (operating theatre, post anaesthetic care unit, and intensive care unit) and timepoints after end-anaesthetic after each of the included studies. Eleven studies collected observations in the operating theatre and used the phrase ‘Emergence Agitation’ to categorise patients who emerged with agitation (Bharadwaj et al., 2022, Cho et al., 2022, Choi et al., 2021, Fei and Yu, 2019, Kang et al., 2019, Kawagoe et al., 2022, Kim et al., 2019, Liu et al., 2022, Meng et al., 2022, Sirivanasandha et al., 2018, Zhang et al., 2020). Six studies named ‘Emergence Agitation’, when reporting data collection after tracheal extubation and patient admission to the post anaesthetic care unit (Cho et al., 2022, Fields et al., 2018, Kawagoe et al., 2022, Kim et al., 2019, Sirivanasandha et al., 2018, Sun et al., 2022). One study (Kong et al., 2021) did not specify the location of data collection.

Fourteen studies used the phrase ‘Emergence Delirium’ for observations collected after transfer from the operating room to the post anaesthetic care unit (Assefa and Sahile, 2019, Awada et al., 2022, Bharadwaj et al., 2022, Braga and Abelha, 2022, Choi et al., 2021, Fei and Yu, 2019, Gu et al., 2022, Huang et al., 2020, Mekonin et al., 2022, Pipanmekaporn et al., 2018, Ramroop et al., 2019, Wiinholdt et al., 2019, Zhang et al., 2021, Zhang et al., 2020), which includes one study (Gu et al., 2022) who used ‘Emergence Delirium’ for first observations taken after patients were admitted to the intensive care unit after tracheal extubation in the operating theatre. Two studies collected first observations in the post anaesthetic care unit and used the umbrella phrase ‘Inadequate Emergence’ (Braga and Abelha, 2022, Wiinholdt et al., 2019) of which ‘Emergence Delirium’ was agitated behaviour and ‘Hypoactive Delirium’ was lethargic, withdrawn behaviour.

Eight studies collected sequential data in first the operating room and then the post anaesthetic care unit (Bharadwaj et al., 2022, Cho et al., 2022, Choi et al., 2021, Fei and Yu, 2019, Kawagoe et al., 2022, Kim et al., 2019, Sirivanasandha et al., 2018, Zhang et al., 2020). Five studies used the term ‘Emergence Agitation’ in both locations (Cho et al., 2022, Kawagoe et al., 2022, Kim et al., 2019, Sirivanasandha et al., 2018, Zhang et al., 2020), three studies used ‘Emergence Agitation’ in the operating room and ‘Emergence Delirium’ in the post anaesthetic care unit (Bharadwaj et al., 2022, Fei and Yu, 2019, Zhang et al., 2020), and one study (Makarem et al., 2020) used the umbrella phrase ‘Inadequate Emergence’ (where agitated behaviour was named ‘Emergence Agitation’ and lethargic, withdrawn behaviour was named Hypoactive Emergence) in both the operating room and the post anaesthetic care unit (Makarem et al., 2020) (Table 1, Table 2, Supplementary Table S1).

### 3.4 Variable descriptors and tools

All studies defined features of anaesthetic emergence agitation by adopting the descriptor of the assessment tool used and then selected a level within that tool to describe the chosen behaviour as agitated. Six different assessment tools were used for first reported data collection. One of these tools had been validated for use by a patient cohort study on adults undergoing emergence from general anaesthetic after cardiac surgery (Riker et al., 2001). None of the other tools used for patient assessment by the studies in this review, including Aono's four Point Scale (Aono et al., 1999), Confusion Assessment Method – Intensive Care Unit (Ely et al., 2001), Nursing Delirium Screening Scale (Gaudreau et al., 2005), 3-Minute Confusion Assessment Method (Marcantonio et al., 2014), and the Richmond Agitation Sedation Scale (Sessler et al., 2002), had undergone a validation study for use in assessment of adult patients recovering from general anaesthetic after surgery (Supplementary Materials M3).

Nine studies used the ten-level Richmond Agitation Sedation Scale (Sessler et al., 2002) grading of greater than or equal to +1 (Restless – Anxious or apprehensive but movements not aggressive or vigorous) to benchmark agitation (Assefa and Sahile, 2019, Braga and Abelha, 2022, Fei and Yu, 2019, Kawagoe et al., 2022, Makarem et al., 2020, Mekonin et al., 2022, Pipanmekaporn et al., 2018, Zhang et al., 2021, Zhang et al., 2020). Fields et al. (2018) chose a baseline of Richmond Agitation Sedation Scale greater than or equal to +3 (Very agitated – pulls on or removes tube(s) or catheter(s) or has aggressive behaviour toward staff) and added to their emergence agitation criteria with the phrase “or administration of haloperidol during PACU [post anaesthetic care unit] stay” (p.1053).

The seven-level Riker-Sedation-Agitation-Scale (Riker et al., 2001) was used by seven studies (Bharadwaj et al., 2022, Cho et al., 2022, Choi et al., 2021, Kang et al., 2019, Kim et al., 2019, Meng et al., 2022, Sirivanasandha et al., 2018), with the benchmark for agitation being greater than or equal to 5 (Agitated – Anxious or mildly agitated, attempting to sit up, calms down on verbal instructions).

The Nursing Delirium Assessment Scale (Gaudreau et al., 2005) was used by three studies (Huang et al., 2020, Ramroop et al., 2019, Wiinholdt et al., 2019), with the benchmark for agitation being ‘Inappropriate behaviour or communication to place and/or for the person, Inappropriate behaviour of 2 = agitated, pulling at devices or climbing out of bed’. The four-point scale assessment tool developed by Aono et al. (1999) was used by two studies (Liu et al., 2022, Sun et al., 2022), where agitated emergence was measured as greater than or equal to 3 (‘Not easily calmed, moderately agitated or restless’). One study (Awada et al., 2022) used the Three-Minute Confusion Assessment Method where a positive score indicated delirium (Marcantonio et al., 2014). One study (Kong et al., 2021) used their own three-point scale, where 0 was calm and cooperative, and 3 was graded agitated behaviour ”moving hands and feet” (p 3642).

Three studies conducted serial observations using different assessment tools to measure initial observations followed by subsequent observation (Fei and Yu, 2019, Mekonin et al., 2022, Zhang et al., 2020). The initial emergence was recorded with the Richmond Agitation Sedation Scale (Sessler et al., 2002) and subsequent observations with the Confusion Assessment Method – Intensive Care Unit (Ely et al., 2001), where a positive result indicated post-operative delirium (Table 1, Table 2, Supplementary Table S1). Refer to Table 1, Table 2, Supplementary Material M3, Supplementary Table S1 for the terminology, data collection points along the patients’ anaesthetic emergence continuum, and brief description of the tools and benchmarks employed by each study.

*Review Question 2. What are the anaesthetic emergence agitation, outcomes, management, and risk factors for patients who have emergency or major surgery, significant pre-operative co-morbidities, or admission to a critical care unit?*

### 3.5 Clinical locations

Outcomes in this review are derived from studies that collected data from the operating theatre or in post anaesthetic care. Two studies (Gu et al., 2022, Kim et al., 2019) reported data from patients admitted to the intensive care unit after elective surgery, but the first data reported was after removal of mechanical ventilation and tracheal extubation. Consequently, these data were grouped and analysed with the other identified studies. Our search did not identify studies that reported anaesthetic emergence data from patients admitted to a critical care setting under general anaesthetic and mechanically ventilated (Table 1, Table 2, Supplementary Table S1). All terms relating to agitated behaviour at anaesthetic emergence will be called ‘emergence agitation’. This includes emergence agitation, emergence delirium and inadequate emergence.

### 3.6 Outcomes

The observational studies reported an emergence agitation occurrence ranging from 510/207,569 (0.25%) (Fields et al., 2018) to 72/208 (34.6%) (Mekonin et al., 2022). Randomised controlled trials reported a control arm occurrence of 7/45 (15.6%) (Cho et al., 2022) to 28/44 (63.6%) (Choi et al., 2021). The intervention arm emergence agitation occurrence ranged from 1/40 (2.5%) (Sirivanasandha et al., 2018) to 13/49 (26.5%) (Choi et al., 2021). Zhang et al. (2021) reported an emergence agitation occurrence of nourished 134/425 (31.5%) to malnourished 205/490 (41.8%) patients (Supplementary Table S2).

Five studies reported dexmedetomidine (an anaesthetic adjunct agent) reduced rates of emergence agitation (Choi et al., 2021, Kang et al., 2019, Kim et al., 2019, Kong et al., 2021, Sun et al., 2022). One study (Sirivanasandha et al., 2018) reported dexmedetomidine had no effect on rates of emergence agitation. Reported side effects of dexmedetomidine included hypotension (Sirivanasandha et al., 2018) and bradycardia (Kang et al., 2019, Kong et al., 2021, Sirivanasandha et al., 2018). Other studies reported that high or low dose glucocorticoids (Awada et al., 2022), and dexamethasone (Cho et al., 2022) did not reduce emergence agitation. Paravertebral regional blocks were more effective than regional nerve blocks after thoracic surgery (Liu et al., 2022) and butorphanol was more effective than placebo (Meng et al., 2022) in reducing emergence agitation after thoracic surgery (Supplementary Table S2).

Ten studies recorded data from post-anaesthetic intervals to measure the duration of emergence, the development of emergence agitation/delirium, emergence delirium where named as sequential, and the subsequent onset of postoperative delirium (Awada et al., 2022, Bharadwaj et al., 2022, Fei and Yu, 2019, Fields et al., 2018, Gu et al., 2022, Huang et al., 2020, Kim et al., 2019, Pipanmekaporn et al., 2018, Zhang et al., 2021, Zhang et al., 2020). Three studies (Fields et al., 2018, Kim et al., 2019, Zhang et al., 2020) reported an association between the onset of emergence agitation and subsequent post-operative delirium (Fields et al., 2018, Kim et al., 2019, Zhang et al., 2020) (Supplementary Table S2).

### 3.7 Clinical features and management

Clinical features requiring management during emergence agitation events were described by Fields et al. (2018). Features included hypertension, violence against staff and self-harm. The self-harm included lacerations and removal of invasive devices.

Specific management of emergence agitation events that occurred in either the operating room or post anaesthetic care unit was reported by four studies (Bharadwaj et al., 2022, Fields et al., 2018, Pipanmekaporn et al., 2018, Sirivanasandha et al., 2018). Management included physical restraint (Fields et al., 2018, Pipanmekaporn et al., 2018, Sirivanasandha et al., 2018), additional staff (Fields et al., 2018) or benzodiazepines (Bharadwaj et al., 2022, Fields et al., 2018, Pipanmekaporn et al., 2018). Additional medications administered included haloperidol (Fields et al., 2018) or opioids (Fields et al., 2018, Pipanmekaporn et al., 2018). Non-pharmacological treatment was described by Sirivanasandha et al. (2018) as “verbal reminding of limits” (2018, p.S95). Extra medical treatment (no other details provided) was reported by Pipanmekaporn et al. (2018) and management of hypertension was reported Fields et al. (2018) (Supplementary Table S2).

### 3.8 Risk factors

Risk factors for emergence agitation were identified in the preoperative, intraoperative and postoperative timeframes. Preoperative risk factors were either modifiable, such as body weight, smoking (Fields et al., 2018), or non-modifiable, such as age and biological gender (Makarem et al., 2020). Intraoperative risk factors included anaesthetic dose, agents and anaesthetic duration longer than two hours (Bharadwaj et al., 2022, Fields et al., 2018), opioids (Bharadwaj et al., 2022, Fields et al., 2018, Makarem et al., 2020, Mekonin et al., 2022), and excessive blood loss (Assefa and Sahile, 2019, Makarem et al., 2020).

Post-operative risk factors included the presence of invasive devices (Fields et al., 2018), post anaesthetic care unit length of stay (Assefa and Sahile, 2019, Fields et al., 2018), post-operative nausea/vomiting (Fields et al., 2018, Kawagoe et al., 2022) and administration of ketamine, opioids, or benzodiazepines (Fields et al., 2018, Makarem et al., 2020) (Supplementary Table S3).

*Review Question 3. What are the gaps in the evidence regarding patients with multiple co-morbid conditions and/or admitted to a critical care unit prior to anaesthetic emergence?*

### 3.8 Exclusion of at-risk patients

Patients with significant risk factors and/or co-morbidities, including patients directly transferred to a critical care unit while still under general anaesthetic and connected to mechanical ventilation, were excluded from all observational studies (Bharadwaj et al., 2022, Braga and Abelha, 2022, Fei and Yu, 2019, Fields et al., 2018, Gu et al., 2022, Huang et al., 2020, Makarem et al., 2020, Mekonin et al., 2022, Pipanmekaporn et al., 2018, Ramroop et al., 2019, Wiinholdt et al., 2019, Zhang et al., 2020) except for Assefa and Sahile (2019). This study reported data for one patient who was admitted to the post anaesthetic care unit with a tracheal tube and mechanical ventilation in situ (Assefa and Sahile, 2019).

All randomised controlled trials (Awada et al., 2022, Cho et al., 2022, Choi et al., 2021, Kang et al., 2019, Kawagoe et al., 2022, Kim et al., 2019, Kong et al., 2021, Liu et al., 2022, Meng et al., 2022, Sirivanasandha et al., 2018, Sun et al., 2022, Zhang et al., 2021) excluded patients with significant co-morbidities and pre-emergence admission to critical care units. Examples of exclusion criteria from enrolment in the study include end-organ failure (Meng et al., 2022), neurological or psychiatric disorder (Bharadwaj et al., 2022) and pregnancy (Gu et al., 2022) (Table 1, Table 3).

**Table 3:** Exclusion Criteria Summary (n = 25).

**Exclusion Criteria**	**Preoperative**										**Postoperative**					
	**Age**	**ASA** **≥ III**	**BMI**	**CVS** **Resp**	**Diab**	**Organ** **dysfn**	**N/P**	**Other**	**Preg** **lactn**	**Subst** **abuse**	**Unst**	**Emerg** **Sx**	**ICU**	**Intbn**	**Other**	**Unst**
Assefa (2019)							√	√					√			
Awada (2022)					√			√	√			√	√		√	
Bharadwaj (2022)							√	√				√	√	√		
Braga (2022)							√					√	√	√		
Cho (2022)	√	√			√	√	√	√	√			√	√			
Choi (2021)	√	√					√	√	√			√	√			
Fei (2019)							√					√	√	√		
Fields (2018)													√	√		
Gu et (2022)		√					√		√			√				
Huang (2020)	√					√	√	√			√	√	√			√
Kang (2019)							√					√	√		√	
Kawagoe (2022)	√	√		√			√	√				√	√		√	
Kim (2019)	√	√	√	√		√	√	√		√		√				
Kong (2021)		√	√	√		√		√				√			√	
Liu et (2022)	√	√	√	√		√	√			√		√	√			
Makarem (2020)		√					√	√				√				
Mekonin (2022)	√					√	√	√			√	√	√			√
Meng (2022)	√	√	√	√		√	√					√	√			
Pipanmekaporn (2018)													√			
Ramroop (2019)							√					√	√	√		
Sirivanasandha (2018)	√	√		√		√		√			√	√	√	√		√
Sun (2022)	√	√	√	√		√	√	√		√		√	√		√	
Wiinholdt (2019)							√	√					√	√		
Zhang (2021)	√	√					√					√	√		√	
Zhang (2020)	√	√					√					√	√	√		

Legend: Age – excluded from any age group over 18 years or older; ASA – American Society of anesthesiologists physical assessment score; BMI – body mass index; CVS – cardiovascular system; Resp – respiratory; Diab – diabetes type 1 or 2; ICU – intensive care unit; Intbn – tracheal tube intubation; Organ dysfn – any organ dysfunction; N/P – neurological/psychiatric disorder; Other – any other exclusion criteria (see Table 1); Preg lacn – patient is pregnant or lactating; Subst abuse – substance *abuse (alcohol, drugs, smoking); Unst – unstable clinical features*

Four studies may have included patients who had undergone emergency surgery (Assefa and Sahile, 2019, Fields et al., 2018, Pipanmekaporn et al., 2018, Wiinholdt et al., 2019), however, their results did not report whether a patient had undergone elective or emergency surgery (Table 1, Table 3).

## 4 Discussion

### 4.1 Key findings

Anaesthetic emergence is a time in which the patient can be vulnerable to significant complications, including emergence agitation (Li and Sun, 2024). This review has detailed outcomes, the timeframe, and points along the anaesthetic emergence continuum in which emergence agitation has been reported.

Risk factors for emergence agitation were identified for patients with significant co-morbid conditions. These risk factors excluded patients from enrolment in most observational and all interventional studies. Review findings included a high level of heterogeneity among studies with respect to research design, timepoint of data collection after end-anaesthetic, characterisation of emergence agitation, and terminology used.

### 4.2 Characterisation

Definitions of emergence agitation varied from objective descriptions of physical behaviour that was highly agitated (Fields et al., 2018), to reporting the patient's subjective level of anxiety and pain (Liu et al., 2022). Anaesthetists, such as Foulds and Dalton (2018) define anaesthetic emergence as physical agitation that occurs in the period between end-anaesthetic and within five minutes after tracheal extubation. This definition contrasts with some studies in our review, such as (Cho et al., 2022, Kawagoe et al., 2022). These studies defined emergence agitation as restlessness that occurred from tracheal extubation to the time of discharge from the postanaesthetic care unit over an hour later (Cho et al., 2022, Kawagoe et al., 2022). In contrast, Tolly et al. (2021) regard agitation in the post anaesthetic care unit as early postoperative delirium. Lee and Sung (2020) report the implications of mis-diagnosing post anaesthetic agitation as emergence agitation instead of early hyperactive postoperative delirium are potentially significant for clinical practice. Emergence agitation is a short-term event that requires immediate management, whereas postoperative delirium is a pathophysiological change in the brain that requires longer-term treatment (Lee and Sung, 2020).

The use of multiple patient assessment tools by the studies in this review added to the challenge to achieve a clear definition of emergence agitation. This challenge was compounded by the different levels within tools used to benchmark patient behaviours that were all named emergence agitation. We have identified that only one of the tools used for patient assessment (Riker et al., 2001) was tested with a cohort of patients who underwent anaesthetic emergence after adult cardiac surgery. Analysing, reconciling and synthesising our review outcomes was further complicated by the variable cohort sizes, and presentation of data collected using tools that were not validated on adult patients undergoing anaesthetic emergence after general surgery, a concern also raised by Greiner and Kremer (2019).

Like Tolly et al. (2021), in combination with inconsistent definitions, tools and cohort sizes, we were unable to synthesise the scoping review outcomes with sufficient confidence to enable a reliable description and definition of emergence agitation after adult general surgery. It is beyond the scope of this review to ascertain what impact, if any, this lack of standardisation may potentially have on clinical practice.

### 4.3 Implications for practice

The patients identified as most at risk for anaesthetic emergence complications by professional bodies, such as the Australian and New Zealand College of Anaesthetists (Australian and New Zealand College of Anaesthetists, 2024) and The Royal College of Anaesthetists and The Difficult Airway Society (The Royal College of Anaesthetists and the Difficult Airway Society, 2011) were not enrolled in interventional studies within our review, and were excluded from most observational studies. The observational studies that assessed risk factors excluded patients admitted to the critical care unit. We did not identify any studies that investigated patients with conditions which routinely led to critical care admission before anaesthetic emergence was initiated.

Hence, outcomes and best practice for managing potential complications of anaesthetic emergence in these patients were not analysed by the studies in our review. We were unable to assess if strategies that are recommended in the non-critical care literature are of benefit. Wei et al. (2021) report that dexmedetomidine, one of the anaesthetic adjuncts shown to reduce the risk of emergence agitation, may not be suitable for patients with significant cardiovascular risk factors. Non-pharmacological measures, such as pre-anaesthetic assessment and individual patient planning for emergence (Lovestrand et al., 2017) may not be possible for a patient who has presented requiring emergency surgery.

Consequently, although risk factors and relevant management strategies were reported by the analysis in the scoping review, none of the studies had investigated these interventions with patients undergoing anaesthetic emergence between critical care admission and tracheal extubation. It is beyond the scope of this review to ascertain what impact, if any, the exclusion of at-risk patients, combined with the lack of standardisation, may potentially have on clinical practice in critical care units.

### 4.4 Evidence gaps

There are several evidence gaps identified by this scoping review. The definition and characterisation of emergence agitation is not standardised regarding nomenclature, time of the emergence after end-anaesthetic, emergence duration, and clinical features. Most tools used to benchmark and classify data obtained by observation of patients were not validated for assessment of anaesthetic emergence behaviours in adult patients after general anaesthetic.

We have identified a lack of evidence regarding outcomes and best practice management for patients with significant risk factors for emergence agitation, including postoperative patients directly admitted to a critical care unit before undergoing anaesthetic emergence and tracheal extubation.

## 5 Limitations

The scoping review mapped the recent literature on anaesthetic emergence agitation in adults who have had general surgery under general anaesthetic. The review has not provided a systematic analysis of incidence and management, or a quality appraisal of included studies but may provide a research opportunity to conduct such a review in the future. It is possible there has been selection bias, since there may be articles that the search strategy did not identify. The interchangeable terminology around the event of anaesthetic emergence may mean that some relevant articles have been missed.

## 6 Conclusion

This review has characterised the emergence continuum. Synthesis of selected studies was thwarted by the heterogenous research design and diverse patient assessment methods at variable emergence continuum points. The variations between studies has highlighted the necessity in the future to reach consensus regarding emergence definition and measurement.

A critical gap was identified regarding recommendations for prevention and management of emergence agitation for patients admitted directly to a critical care unit. Further research is recommended to include the patients most at-risk of emergence agitation.

## Funding sources

None

## CRediT authorship contribution statement

**Meredith Heily:** Writing – review & editing, Writing – original draft, Visualization, Project administration, Methodology, Investigation, Formal analysis, Data curation, Conceptualization. **Marie Gerdtz:** Writing – review & editing, Validation, Supervision, Methodology. **Rebecca Jarden:** Writing – review & editing, Validation, Supervision, Methodology. **Yen Yap:** Writing – review & editing, Validation, Supervision, Methodology. **Rinaldo Bellomo:** Writing – review & editing, Validation, Supervision, Methodology.

## Declaration of competing interest

None

## Appendix Supplementary materials

Image, application 1

Image, application 2

Image, application 3

Image, application 4
