# Supplementary material for: Anaesthetic emergence agitation in adults following general surgery: A scoping review
Source: Int J Nurs Stud Adv. 2025 Mar 18;8:100320. doi: 10.1016/j.ijnsa.2025.100320 (PMC11984576; doi:10.1016/j.ijnsa.2025.100320)
Supplement: Supplementary file 1 [file mmc1.docx]

**Supplementary Materials M3.**

**Assessment Tools with scales**

This is a brief description of the tools used to assess the patients and report outcomes, within the scoping review.

**3-Minute Diagnostic Interview for CAM-defined Delirium (3D-CAM) (Marcantonio et al., 2014)**

A four-feature test of conscious patients to test if they are confused or orientated. A positive result indicates delirium.

Features: 1 = acute change/fluctuating course, 2 = inattention, 3 = disorganised thinking, 4 = altered level of consciousness

Validation study cohort (n = 201patients): Patients 75 years old, admitted to a general medical or geriatric services ward. No patient was recovering from a general anaesthetic.

**Aono’s Four Point Score (4PS)** (Aono et al., 1999)

Four-point scale measuring patients’ behaviour after they are alert and conscious.

Scale: 1 = calm, 2 = not calm but could easily be calmed, 3 = not easily calmed, moderately anxious, 4 = excited or disorientated

Validation study cohort (n = 110 patients): Patients (boys) aged 3 – 6 years old emerging from anaesthetic after minor urological surgery.

**Confusion Assessment Method – Intensive Care Unit (CAM-ICU)** (Ely et al., 2001)

A four-feature test of conscious patients to test if they are confused or orientated. A positive result indicates delirium.

Physical responses e.g. appropriate use of thumbs up/thumbs down, are used to guide assessment. Outcomes are either positive or negative for delirium.

Features: 1 = acute change/fluctuating course, 2 = inattention, 3 = disorganised thinking, 4 = altered level of consciousness

Validation study cohort (n = 96 patients): mechanically ventilated patients in an adult medical and coronary intensive care unit. Some were sedated, none were recovering from a general anaesthetic.

**Nursing Delirium Screening Scale (Nu-DESC)** (Gaudreau et al., 2005)

A delirium scale rating each of five domains from 0 to 2 based on the presence and intensity of each symptom. Individual domain ratings are added together to obtain a total score out of 10.

Domains: I = disorientation, II = inappropriate behaviour, III – inappropriate communication, IV = illusions/hallucinations, V = psychomotor retardation

Tool validation study cohort (n = 146 patients): patients admitted to a hemato-oncological medicine unit. No patient was recovering from a general anaesthetic.

**Pediatric Anesthesia Emergence Delirium Scale** (Sikich and Lerman, 2004)

A five-level scale with five domains. Domains assessing children include eye contact, actions, awareness of surroundings, level of restlessness and level of inconsolability.

Scale part 1. Domains (Eye contact, actions and awareness of surroundings): 4 = not at all, 3 = just a little, 2 = quite a bit, 1 = very much, 0 = extremely.

Scale part 2. Domains (Restless and inconsolable): 0 = not at all, 1 = just a little, 2 = 1uite a bit, 3 = very much, 4 = extremely

Validation study cohort (n = 50 patients): Conscious children in the postanaesthetic care unit recovering from mixed surgical procedures under general anaesthetic.

**Richmond Agitation Sedation Scale (RASS)** (Sessler et al., 2002)

An eleven-point scale measuring patient response from deeply comatose to dangerously agitated.

Scale: -5 = unarousable, -4 = deep sedation, -3 = moderate sedation, -2 = light sedation, -1 = drowsy, 0 = calm, +1 = restless, +2 = agitated, +3 = very agitated, + 4 = combative

Tool validation study cohort (n = 172 patients): mixed intensive care unit patients (medical, surgical, cardiac). 91 patients were sedated, 67 were mechanically ventilated. It was not reported if the sedated patients were recovering from general anaesthetic.

**Riker Agitation Sedation Scale (RSAS)** (Riker et al., 2001)

A seven-point scale measuring response from deeply comatose to dangerously agitated.

Scale: 1 = unarousable, 2 = very sedated, 3 = sedated, 4 = calm, cooperative, 5 = agitated, 6 = very agitated, 7 = dangerous agitated.

Tool validation study cohort (n = 39 patients): sedated, mechanically ventilated patients in ICU recovering from general anaesthetic after adult cardiac surgery. The number of agitated or non-agitated patients on emergence was not reported. Results compared the Bispectral Index (BIS) monitor of depth of anaesthesia with the RSAS scale to compare the patient level of sedation and agitation. Results reported the RSAS correlated with the BIS monitor regarding patient level of sedation/wakefulness/agitation.

**Three-point Scale** (Kong et al., 2021)

A three-point agitation scale.

Scale: 0 = calm and cooperative, 1 = complained of discomfort when questioned, 2 = patient frequently complains of discomfort, 3 = behavioural responses such as moving hands and feet.

Validation study – scale not validated. Reference used by article not applicable to the three-point scale (referenced article described a surgical technique, did not refer to anaesthesia).

References

Aono, J., Mamiya, K. & Manabe, M. 1999. Preoperative anxiety is associated with a high incidence of problematic behavior on emergence after halothane anesthesia in boys. *Acta Anaesthesiologica Scandinavica,* 43**,** 542-4.

Ely, E. W., Inouye, S. K., Bernard, G. R., Gordon, S., Francis, J., May, L., Truman, B., Speroff, T., Gautam, S., Margolin, R., Hart, R. P. & Dittus, R. 2001. Delirium in mechanically ventilated patients: validity and reliability of the confusion assessment method for the intensive care unit (CAM-ICU). *JAMA: Journal of the American Medical Association,* 286**,** 2703-2746.

Gaudreau, J., Gagnon, P., Harel, F., Tremblay, A. & Roy, M. 2005. Fast, systematic, and continuous delirium assessment in hospitalized patients: the Nursing Delirium Screening Scale. *Journal of Pain & Symptom Management,* 29**,** 368-375.

Kong, Y., Bai, Z., Chen, L. & Yao, Y. 2021. Effects of different doses of dexmedetomidine in patients with colorectal cancer. *Acta Medica Mediterranea,* 37**,** 3641-3646.

Marcantonio, E. R., Ngo, L. H., O'connor, M., Jones, R. N., Crane, P. K., Metzger, E. D. & Inouye, S. K. 2014. 3D-CAM: derivation and validation of a 3-minute diagnostic interview for CAM-defined delirium: a cross-sectional diagnostic test study. *Annals of Internal Medicine,* 161**,** 554-561.

Riker, R., Fraser, G. L., Simmons, L. & Wilkins, M. 2001. Validating the Sedation-Agitation Scale with the Bispectral Index and Visual Analog Scale in adult ICU patients after cardiac surgery. *Intensive Care Medicine,* 27**,** 853-858.

Sessler, C., Gosnell, M., Grap, M. J., Brophy, G., O'neal, P., Keane, K., Tesoro, E. & Elswick, R. 2002. The Richmond Agitation-Sedation Scale: validity and reliability in adult intensive care unit patients. *American Journal of Respiratory and Critical Care Medicine,* American Journal of Respiratory and Critical Care Medicine**,** 1338-44.

Sikich, N. & Lerman, J. 2004. Development and psychometric evaluation of the pediatric anesthesia emergence delirium scale. *Anesthesiology,* 100**,** 1138-45.
