# Supplementary material for: Anaesthetic emergence agitation in adults following general surgery: A scoping review
Source: Int J Nurs Stud Adv. 2025 Mar 18;8:100320. doi: 10.1016/j.ijnsa.2025.100320 (PMC11984576; doi:10.1016/j.ijnsa.2025.100320)
Supplement: Supplementary file 2 [file mmc2.docx]

| Supplementary Table. S1 Study observation timepoints, assessment tools & definitions (n = 25) | | | | | |
| --- | --- | --- | --- | --- | --- |
| **STUDY** | **TOOL** | **Name** | **Time**  **point** | **Objective**  **Signs** | **Patient Subjective** |
| Assefa & Sahile (2019) | RASS > +1 | ED | 5 | Restless, movements not aggressive or vigorous | Anxious, apprehensive |
| Awada et al (2022) | 3D-CAM positive | ED | 6, 8 | Restless, movements not aggressive or vigorous | Anxious, apprehensive |
| Bharadwaj (2022) | RSAS > 5 | EA then ED | 5,6,7 | Mildly agitated, attempting to sit up, calms down on verbal instruction | Anxious |
| Braga & Abelha (2022) | RASS > +1 | IE | 5, 8 | Restless, movements not aggressive or vigorous | Anxious, apprehensive |
| Cho (2022) | RSAS > 5 | EA | 1, 2, 3, 4, 5, 6, 7, 8 | Mildly agitated, attempting to sit up, calms down on verbal instruction | Anxious |
| Choi et al (2021) | RSAS > 5 | EA | 3, 5 | Mildly agitated, attempting to sit up, calms down on verbal instruction | Anxious |
| Fei & Yu (2019) | RASS > +1 | EA then ED | 3, 5,6,7,8 | Restless, movements not aggressive or vigorous | Anxious, apprehensive |
| Fields et al (2018) | RASS > +3 | EA (retro) | 5,6,7,8 | Severe agitation - pulls or attempts to pull at tubes OR haloperidol administration | Nil |
| Gu et al (2022) | RASS > +1 | ED | 5 | Restless, movements not aggressive or vigorous | Anxious, apprehensive |
| Huang et al (2020) | Nu-DESC > 2 | ED | 6,7 | Moderately severe | Hallucinations, |
| Kang et al (2019) | RSAS > 5 | EA (retro) | 4 | Restlessness, agitation, noncooperation, confusion, disorientation, violent behaviour | Nil |
| Kawagoe et al (2022) | RASS > +1 | EA | 3,4,5,6,7 | Restless, movements not aggressive or vigorous | Anxious, apprehensive |
| Kim et al (2019) | RSAS > 4 | EA | 3,4,5,6,7,8 | Mildly agitated, attempting to sit up, calms down on verbal instruction) | Anxious |
| Kong et al (2021) | 3PS > 2 | EA | 5 | Moves hands and feet | Complained of discomfort |
| Liu et al (2022) | Aono’s 4PS > 3 | EA | 1,2,3 | Not easily calmed, moderately agitated or restless | Nil |
| Makarem et al (2020) | RASS > +1 | IE/EA | 6 | Restless, movements not aggressive or vigorous | Anxious, apprehensive |
| Mekonin et al (2022) | RASS > +1 | ED | 5,6 | Responds to physical stimuli. | Anxious, apprehensive |
| Meng et al (2022) | RSAS > 4 | EA | 4 | Mildly agitated, attempting to sit up, calms down on verbal instruction | Nil |
| Pipanmekaporn 2018) | RASS > +1 | ED | 5,6,7,8 | Restless, movements not aggressive or vigorous | Anxious, apprehensive |
| Ramroop et al (2019) | Nu-DESC > 2 | ED | 6 | Moderately severe | Hallucinations |
| Sirivanasandha et al (2018) | RSAS > 5 | EA | 2,3,4,6 | Mildly agitated, attempting to sit up, calms down on verbal instruction | Anxious |
| Sun et al (2022) | 4PS > 3 | EA | 5,6 | Not easily calmed, moderately agitated or restless | Nil |
| Wiinholdt et al (2019 | Nu-DESC > 2 | IE | 5,6,8 | Moderately severe | Hallucinations |
| Zhang (2021) | RASS > +1 | ED | 5,6 | Restless, movements not aggressive or vigorous | Anxious |
| Zhang et al (2020) | RASS > +1 | ED | 3,4,6 | Restless, movements not aggressive or vigorous  CAM-ICU - positive - confused, inappropriate answers | Hallucinations |
| *Data collection timepoints: 1 – End-anaesthetic: 2 – pre-extubation; 3 – extubation; 4-five minutes after extubation; 5 – O/A PACU; 6 – PACU at 30 minutes; 7 – PACU 60 minutes; 8 – ward assessment*  *Legend: 3D-CAM – three-dimensional confusion assessment method; 3PS – three-point scale; Aono’s 4PS – Aono’s four-point scale; EA – emergence agitation; ED – emergence delirium; IE – inadequate emergence; Nu-DESC – Nursing Delirium Screening Scale; RASS – Richmond Agitation Sedation Scale; RSAS – Riker Sedation Agitation Scale* | | | | | |
