# Supplementary material for: Anaesthetic emergence agitation in adults following general surgery: A scoping review
Source: Int J Nurs Stud Adv. 2025 Mar 18;8:100320. doi: 10.1016/j.ijnsa.2025.100320 (PMC11984576; doi:10.1016/j.ijnsa.2025.100320)
Supplement: Supplementary file 3 [file mmc3.docx]

| Supplementary Table S2 Reported outcomes (n = 25) | | | |
| --- | --- | --- | --- |
| **Location** | **EA/Total patients (%)** | | **Outcomes** |
|  | **No Intervention** | **Intervention** |  |
| **OT Pre-Extubation** | | | |
| Cho (2022) | 7/45 (15.6%) | 3/45 (6.7%) | Dexamethasone - no effect |
| Choi (2021) | 28/44 (63.6%) | 10/44 (22.7%) | Dexmed reduced EA &  postoperative pain |
| Fei (2019) | 69/380 (18.2%) | Nil | EA observed |
| Kawagoe (2022) | 20/40 (50.0%) | 4/40 (10.0%) | Dexmed reduced EA |
| Liu (2022) | 15/48 (31.3%) | 6/49 (12.2%) | More EA in desflurane group |
| Sirivana-  sandha (2018) | 20/47 (42.6%) | 13/49 (26.5%) | No EA difference b/w Dexmed  & placebo groups. |
| Zhang, Y. (2020) | 99/915 (10.8%) | Nil | EA linked with ED & POD,  RF identified |
| **OT Post-Extubation** | | | |
| Bharadwaj (2022) | 9/320 (2.8%) | Nil | RF identified |
| Gu (2022) | 22/618 (3.6%) | Nil | EA observed |
| Kim JA (2019) | 21/60 (35.0%) | 8/60 (13.3%) | Dexmed reduced EA,  but not POD |
| Kong (2021) | nr/60 | nr/120 | Dexmed reduced EA |
| Makarem (2020) | 158/1,136 (13.9%) | Nil | RF Identified |
| Meng (2022) | 75/306 (24.5%) | 29/296 (9.8%) | Butorphanol reduced EA |
| **Post Anaesthetic Care Unit** | | | |
| Assefa (2019) | 97/306 (31.7%) | Nil | EA observed |
| Awada (2022) | 6/27 (22.2%) | 5/26 (19.2%) | EA observed  No difference b/w  high or low dose steroids |
| Braga (2022) | 12/148 (8.1%) | Nil | EA observed |
| Fields (2018) | 510/207,569 (0.25%) | Nil | RF identified, EA features reported: agitation, hypertension, self-harm, removal of invasive devices, violence against staff Management described  EA linked to POD |
| Huang (2020) | 58/168 (34.5%) | Nil | EA observed |
| Kang (2019) | 248/1,654 (15.0%) | 89/814  (10.9%) | Dexmed reduced EA Intraoperative bradycardia |
| Tesfaye Mekonin (2022) | 72/208 (34.6%) | Nil | EA with GA observed |
| Pipanmekaporn (2018) | 17/2,206 (0.8%) | Nil | EA management reported |
| Ramroop (2019) | 49/368 (13.3%) | Nil | EA observed |
| Sun (2022) | 9/40 (22.5%) | 1/40 (2.5%) | Dexmed reduced EA |
| Wiinholdt (2019) | 103/1,000 (10.3%) | Nil | IE observed - EA not differentiated from delirium analysis |
| Zhang,Y. (2020) | 134/425 (31.5%) | 205/490 (41.8%) | EA observed |
| *Legend: b/w – between; Dexmed – dexmedetomidine; EA – emergence agitation; ED – emergence delirium; GA – general anaesthetic; IE – Inadequate Emergence; n/a – not applicable; nr – not reported; OT – operating theatre; POD – postoperative delirium; RF – Risk Factors* | | | |
