# Supplementary material for: Anaesthetic emergence agitation in adults following general surgery: A scoping review
Source: Int J Nurs Stud Adv. 2025 Mar 18;8:100320. doi: 10.1016/j.ijnsa.2025.100320 (PMC11984576; doi:10.1016/j.ijnsa.2025.100320)
Supplement: Supplementary file 4 [file mmc4.docx]

| Supplementary Table S3 Risk Factors | | | |
| --- | --- | --- | --- |
| **Study** | **Pre-operative** | **Intra-operative** | **Post-operative** |
| Assefa (2019) | n/r | Excessive blood loss | PACU LOS |
| Bharadwaj (2022) | Elderly, neurological or cognitive impairment, ASA > III, low education level | Anaesthetic type/ dose/ duration, Opioids | SpO_2_ < 92% for > 4 min |
| Fields (2018) | Substance abuse/smoking, anxiety/psychiatric problems, neurological/cognitive impairment, obese, falls risk | Anaesthetic type, dose, duration,  Opioids, BZD, invasive devices & tubes, delayed extubation | Invasive devices, urinary symptoms, PACU LOS, PONV, respiratory,  Ketamine/opioids/BZD |
| Huang (2020) | Elderly | n/r | n/r |
| Kawagoe (2022) | n/r | n/r | PONV |
| Makarem (2020) | Anxiety/psychiatric problems, neurological/cognitive impairment, hypertension, divorced, male gender | Opioids, Excessive blood loss | Ketamine/opioids/BZD |
| Tesfaye Mekonin (2022) | Elderly, ethnicity, | Opioids, anticholinergics | n/r |
| Zhang, Y. (2020) | Advanced age | n/r | Higher pain scores. |
| *Legend: Anticholinergics – anticholinergic agents; ASA – American Society of Anesthesiologists; BZD – benzodiazepines; HT – hypertensives; LOS – length of stay; n/r – not reported; PACU – postanaesthetic care unit; PONV – postoperative nausea & vomiting; SpO_2_ – oxygen saturation* | | | |
